# Clinical Relevance of Contact Allergy to Gold Sodium Thiosulphate in Fibromyalgia

**DOI:** 10.2340/actadv.v105.42463

**Published:** 2025-03-27

**Authors:** Katharine HOPKINS, Anarita ANTELMI, Jakob DAHLIN, Karin OLSSON, Cecilia SVEDMAN, Jacqueline ÅSTRAND, Peter OLSSON, Magnus BRUZE

**Affiliations:** 1 Department of Occupational and Environmental Dermatology, Lund University, Skåne University Hospital, Malmö, Sweden; 2 Department of Dermatology, Lund University, Skåne University Hospital, Malmö, Sweden; 3 Department of Clinical Sciences, Malmö, Rheumatology, Lund University, Malmö, Sweden; 4 Department of Rheumatology, Skåne University Hospital, Malmö, Sweden, Sweden

**Keywords:** allergic contact dermatitis, fibromyalgia, gold sodium thiosulphate, patch tests, visual analogue scales

## Abstract

Fibromyalgia is a chronic syndrome characterized by pain, fatigue, and cognitive disturbances. The increased prevalence of contact allergy to gold in individuals with fibromyalgia when compared with the general population has previously been described. Gold contact allergy can manifest as a systemic contact dermatitis, with cutaneous and extracutaneous manifestations presenting upon systemic administration of gold. This study aimed to establish whether gold allergy is of significance in the fibromyalgia population. Prior to patch testing with the Swedish baseline series and an extended dental series, 119 females with fibromyalgia answered questionnaires including details of past medical history, dental history, and previous cutaneous and mucous membrane intolerance to metals. Prevalence of allergy to gold sodium thiosulphate (2.0% and 5.0%) was 33.6% (40 individuals). There was a statistically significant overrepresentation of gold allergy among individuals who experienced cutaneous symptoms upon direct contact with gold (*p* = 0.010). Contact allergy to gold was more frequent among patients with oral symptoms (*p* = 0.024). This study demonstrates concordance between reported cutaneous symptomatology related to gold exposure and gold allergy in the fibromyalgia population. Whether individuals with oral symptoms and gold allergy have objective oral clinical findings and relevant gold exposure is the focus of ongoing study.

## SIGNIFICANCE

People with gold allergy do not usually experience problems with gold in contact with the skin (such as with jewellery). However, when used internally, such as with gold dental restorations, gold can cause symptoms in individuals with gold allergy. In this study we show that individuals who have fibromyalgia and gold allergy experience skin problems when wearing gold jewellery and individuals with gold allergy report more oral problems. Fibromyalgia may make patients more susceptible to problems related to gold allergy, through potential increased exposure to gold, or other factors such as poor oral health.

Fibromyalgia (FM) is a common (prevalence 2% in USA), chronic syndrome with a hitherto unknown aetiology, characterized by widespread pain, fatigue, sleep and cognitive disturbances (1, 2). It is currently accepted that individuals with FM have an exaggerated response to noxious stimuli that is mediated by the central nervous system, but a specific trigger for this response is yet to be identified (3, 4). The hunt for this purported trigger is especially challenging due to the heterogeneous presentation of the condition, but a genetic predisposition coupled with an inciting or aggravating environmental event such as infection has been proposed (4, 5).

Contact allergy in the FM population has, until now, not been explored in detail, with a handful of studies reporting on prevalence of metal allergies, particularly nickel allergy, in mixed FM/chronic fatigue syndrome groups (6–9). It has been suggested that the presence of metals in, for example, dental restorations could be a trigger or contributor to symptoms in FM (6–8).

Gold contact allergy is common, with frequencies of positive test reactions to gold sodium thiosulphate (GSTS) 0.5% of approximately 10% in dermatitis patients (10–12). It has been established that gold contact allergy can manifest as a systemic contact dermatitis, with cutaneous and extracutaneous manifestations presenting upon systemic administration of gold to gold-allergic individuals (13–16). It has also been demonstrated that there is a long-term low systemic release of gold from dental restorations and that contact allergy to gold is correlated to dental gold (17, 18). Allergy to dental materials including gold has been implicated in oral conditions including lichenoid lesions (19–21).

This questionnaire study is part of a larger project aimed at exploring contact allergy in FM. Initial surveys assessing prevalence of contact allergy in this group have already been reported (22, 23), where it was found that the rate of contact allergy to GSTS in individuals with FM is significantly higher than in the general population (22). Considering these findings, and our knowledge of the characteristics of gold as an allergen as already outlined, we hypothesize that manifest gold contact allergy may be of clinical importance in the FM population. The aims of the current study were to investigate the exposure and symptomatology to (a) cutaneous gold and (b) dental gold in individuals with FM and gold allergy.

## MATERIALS AND METHODS

### Participants

Volunteers were recruited from local branches of the Swedish Fibromyalgia Association (*Fibromyalgiförbundet*). Detailed description of the recruitment process has previously been published (23). The majority of participants had not previously been patch tested. There were 119 females (mean age 59.5 years, standard deviation 11.9) and 1 male (59 years). The male participant has been excluded from our analysis of patch testing and questionnaire answers for ease of analysis. The questionnaire was answered by 118 participants.

### Questionnaires

To avoid bias and ascertain any clinical significance and relevance of positive patch test reactions volunteers answered questionnaires on the first visit, prior to patch testing (Appendix S1) (24). Questions included: (A) medical history including FM diagnosis, medications, allergies, and rheumatological, psychiatric, and dermatological conditions; (B) dental history including gold restorations, previous cutaneous and mucous membrane intolerance to metals, presence of piercings, intolerance to fragrances; (C) visual analogue scale (VAS) grading (0–100 mm) of FM severity over the past week in terms of mood, fatigue, and pain (25).

### Patch testing

All volunteers underwent patch testing with the Swedish baseline series and an extended dental series consisting of 47 substances including metals, acrylates, fragrances, and preservatives (Appendix S2). A detailed summary of the test materials and methodology has previously been described (23). All participants underwent 2 test readings. The first reading took place at either day (D) 3 or 4 after the test application and the second at D7. Regarding gold, individuals with a “doubtful reaction” upon the first test reading received additional testing with GSTS 5.0% w/w in petrolatum. Late reactions (those occurring after the second reading on D7) to both GSTS 2.0% and GSTS 5.0% were regarded as positive reactions.

### Statistical analysis

A two-sided Fisher’s exact test was used for the analysis of questionnaire answers between groups. For the analysis of VAS scores, the Mann–Whitney *U* test was used, because the data were non-normally distributed. Results with a *p*-value < 0.05 were considered significant.

## RESULTS

Among the 119 female patients assessed for contact allergy, 30 (25.2%) patients demonstrated allergy to GSTS 2.0% (**Table I**). When including allergy to GSTS 5.0% and late reactions the number rises to 40 (33.6%) with 8 reactions to GSTS 5.0% and 2 late reactions (to both GSTS 2.0% and GSTS 5%). Nickel allergy was observed in 33 participants (27.5%).

**Table I:** Number of individuals with and degree of reaction to gold sodium thiosulphate (GSTS)

Grade of gold contact allergy	Number
GSTS 2.0% +	15
GSTS 2.0% ++	9
GSTS 2.0% +++	6
GSTS 5.0%	8
Late reaction	2
Total	40

+ Weak positive reaction,

++ strong positive reaction,

+++ extreme positive reaction.

**Table II** presents the distribution of questionnaire answers. Of 118 respondents, 30 (25.4%) had experienced skin symptoms upon skin contact with gold. Over one-third of respondents (39.8%) reported oral symptoms and 35 individuals (29.7%) reported current or previous dental gold restorations.

**Table II:** Questionnaire responses

Question	No. (%) of answers		
	Yes	No	No answer given
1. Do you experience, or have you previously experienced symptoms such as itching, redness, or swelling from direct skin contact with gold objects (e.g., jewellery)?	30 (25.4)	72 (61)	16 (13.6)
2. Do you have any oral symptoms such as itching, stinging, redness, or ulcers?	47 (39.8)	64 (54.2)	7 (5.9)
3. Do you have or have you previously had any dental gold?	35 (29.7)	77 (65.3)	6 (5.1)
4. Answer “yes” to both questions 2 and 3	14 (11.9)		
5. Do you experience, or have you previously experienced symptoms such as itching, redness, or swelling from direct skin contact with non-precious metals (e.g., jewellery)?	82 (69.5)	24 (20.3)	12 (10.2)
6. Do you have eczema, or did you have eczema in childhood?	37 (31.4)	60 (50.8)	21 (17.8)
7. Do you smoke?	14 (11.8)	104 (88.2)	0

Figures for gold allergy and questionnaire answers are given in **Table III**. There was a statistically significant overrepresentation of gold allergy among individuals who experienced cutaneous symptoms upon direct contact with gold (*p* = 0.010). Contact allergy to gold was also more frequent among patients with oral symptoms (*p* = 0.024). Reported symptoms arising from cutaneous contact with non-precious metals were overrepresented in those with gold allergy, although this difference did not reach statistical significance (*p* = 0.051).

**Table III:** Questionnaire answers according to gold and nickel allergy

Question	No. (%) of answers according to gold contact allergy status					No. (%) of answers according to nickel contact allergy status				
	Gold contact allergy+		Gold contact allergy –		*p*-value	Nickel contact allergy+		Nickel contact allergy –		*p*-value
	Yes	No	Yes	No		Yes	No	Yes	No	
1. Do you experience, or have you previously experienced symptoms such as itching, redness, or swelling from direct skin contact with gold objects (e.g., jewellery)?	16 (47.1)	18 (52.9)	14 (20.6)	54 (79.4)	**0.010**	8 (28.6)	20 (71.4)	22 (29.7)	52 (70.3)	1
2. Do you have any oral symptoms such as itching, stinging, redness, or ulcers?	21 (58.3)	15 (41.7)	26 (34.7)	49 (65.3)	**0.024**	16 (53.3)	14 (46.7)	31 (12.8)	50 (61.7)	0.20
3. Do you have or have you previously had any dental gold?	11 (28.9)	27 (71.1)	24 (32.4)	50 (67.6)	0.83	–	–	–	–	–
4. Answer “yes” to both questions 2 and 3	7 (35)	13 (65)	7 (17.5)	33 (82.5)	0.20	–	–	–	–	–
5. Do you experience, or have you previously experienced symptoms such as itching, redness, or swelling from direct skin contact with non-precious metals (e.g., jewellery)?	32 (88.9)	4 (0.11)	50 (71.4)	20 (28.6)	0.051	29 (90.6)	3 (9.4)	53 (71.6)	21 (28.4)	**0.042**
6. Do you have eczema, or did you have eczema in childhood?	13 (41.9)	18 (58.1)	24 (36.4)	42 (63.6)	0.66	8 (28.6)	20 (71.4)	29 (42)	40 (60)	0.25
7. Do you smoke?	5 (12.5)	35 (87.5)	9 (13)	69 (87)	0.21	–	–	–	–	–

Significant values in bold.

Comparisons between the groups with and without nickel allergy are presented in Table III. Significantly more patients with nickel allergy reported cutaneous symptoms upon exposure to non-precious metals than those without nickel allergy (90.6% vs 71.6%; *p* = 0.042).

**Table IV** gives the distribution of VAS scores in relation to different questionnaire answers and clinical parameters. Individuals with oral symptoms scored higher on each scale but only statistically significantly concerning general malaise (*p* = 0.020). Individuals with gold allergy and oral symptoms scored significantly higher than those with gold allergy without oral symptoms across all VAS measurements (*p* < 0.001).

**Table IV:** Results of visual analogue scale (VAS) analysis

Item	Overall VAS (*n*)	Oral symptoms (*n*)	No oral symptoms (*n*)	*p-*value	Gold allergy (*n*)	No gold allergy (*n*)	*p-*value	Gold allergy		*p-*value
								Oral symptoms + (*n*)	Oral symptoms – (*n*)	
Mean VAS pain	61.78 (117)	64.79 (47)	58.26 (65)	0.081	64.50 (40)	60.37 (78)	0.48	69.86 (21)	55.8 (15)	**< 0.001**
Mean VAS general malaise	61.44 (117)	66.51 (47)	57.30 (64)	**0.020**	67.20 (35)	59.64 (74)	0.24	72.91 (22)	51.07 (14)	**< 0.001**
Mean VAS tiredness	66.34 (117)	69.45 (47)	64.28 (68)	0.061	71.22 (36)	65.13 (76)	0.34	75.83 (21)	61.44 (16)	**< 0.001**

Significant values in bold.

## DISCUSSION

The significant findings in this study – namely concordance between reported cutaneous symptomatology related to gold exposure and subsequent positive test reaction to GSTS, and the apparent correlation between oral symptoms, gold allergy, and increased general symptomatology within the FM population – are of particular interest given the hitherto challenging pathway in the investigation and establishment of gold as a contact allergen.

Positive patch test reactions to gold salts are common (10–23% prevalence with GSTS 0.5%) (10–13) but correlation between positive tests and cutaneous symptoms has been more difficult to identify (27, 28). In the research setting, as in the current study, pre-testing questionnaires regarding exposure and symptomatology are used to attempt to limit any bias in analysis of relevance of positive test readings. Several studies prior to this current work have followed this approach with differing results. The first study to use a pretest questionnaire did not find a correlation between cutaneous symptomatology related to gold exposure and subsequent positive patch test for gold. These findings were replicated in a subsequent multicentre study, which also found a higher level of GSTS allergy in control patients than dermatitis patients (29). However, others have demonstrated findings similar to those in this study (10).

Possible explanations for the previously described poor correlation between contact allergy to gold and clinical relevance include a historic lack of consideration of the special qualities of gold as a contact allergen, including a tendency to late and persistent positive reactions without active sensitization (30). In this study, late (> 7 days) reactions to GSTS and reactions to higher test concentrations of GSTS were included. Previously, reactions to gold jewellery were regarded as an irritant contact dermatitis or a concealed contact allergy to other allergens such as nickel due to the presence of nickel in gold alloys of less than 24 carats (31). In the current study there was no difference in prevalence of atopic eczema between those who had gold allergy and those who were not gold-allergic (*p* = 1). If gold reactions were indeed irritant, one would expect to see more reactions in those with a history of atopic eczema and therefore increased propensity to irritant reactions. The reporting of symptoms relating to contact with non-precious metals was significantly higher in those with nickel allergy (*p* = 0.042), while there was no difference in symptomatology related to cutaneous reactions to gold between those with and without nickel allergy (*p* = 1). This strengthens the assertion that there is a correlation between symptomatology and gold contact allergy in this group, as well as eliminating the possibility of symptoms attributed to nickel being a confounder.

There is more convincing evidence of relevant reactions when gold-allergic individuals are exposed to gold through routes other than the skin. Extracutaneous tissues such as the oral mucosa may represent a favourable environment for haptenization. Elemental gold is inert, and conditions on the skin do not promote ionization and thus haptenization (32, 33), meaning that gold release is not sufficient to reach the threshold required to elicit a contact dermatitis. However, gold is a common relevant allergen in the case of medical implants. Positive test reactions are more common in patients with gold dental restorations (18, 34), and correlation with objective clinical findings such as oral lichenoid lesions is also suggested (21, 35). Limited studies have demonstrated an improvement in oral symptoms upon removal of gold-containing restorations in individuals with gold allergy (6, 36). Gold-plated coronary stents can be a route to sensitization to gold, and in-stent stenosis occurs more frequently in gold-allergic patients with gold-plated coronary stents (37). In the current study, a significantly higher number of individuals with gold allergy also reported the presence of oral symptoms (*p* = 0.024), although there did not appear to be a relationship between the presence of gold dental restorations and gold allergy.

Our previously published studies performed on this FM cohort demonstrate that the pattern of contact allergy in this group appears to be specific for substances that can cause sensitization via the oral route, namely gold, acrylates, and certain fragrances that are also used as flavouring substances (22, 23). The prevalence of gold allergy in the FM group was 33.6%, a frequency previously demonstrated in patients with chronic systemic exposure to gold via gold-plated coronary stents (22, 37). Together with the findings in the current study, we can conclude that gold does appear to be a significant allergen in FM. However, further pieces of the puzzle remain to be explored, including gold exposure in these individuals and whether individuals with FM have any behavioural patterns or specific cutaneous characteristics that increase the propensity to allergic contact dermatitis on exposure to gold. An interesting finding in this study was the high prevalence of atopic dermatitis of 31.6% in the FM study population. Atopy in FM has not been studied extensively but pain syndromes have been described as comorbidity in atopic disease (38). Patients with FM have high levels of subjective oral symptoms (39, 40), but the presence of clinical oral lesions has not been shown to be increased when compared with controls. Further study of the current cohort of FM individuals is in progress to assess whether positive clinical findings in terms of stomatitis or lichenoid reactions are associated with relevant gold exposure (gold dental restorations).

Potential weaknesses in this study include the small sample size and the lack of objective assessment as to the presence of dental gold and other metals. This will be addressed in the current ongoing study, which will assess whether the findings of this survey are reproducible.

In conclusion, gold allergy appears to be of clinical relevance in patients with FM. Further work is required to assess the precise exposure profile of this group and whether gold allergy has any further significance in the aetiology of the condition.

## Supplementary Material

Clinical Relevance of Contact Allergy to Gold Sodium Thiosulphate in Fibromyalgia

## References

[1] Vincent A, Lahr BD, Wolfe F, Clauw DJ, Whipple MO, Oh TH, et al. Prevalence of fibromyalgia: a population-based study in Olmsted County, Minnesota, utilizing the Rochester Epidemiology Project. Arthritis Care Res (Hoboken) 2013; 65: 786–792. 10.1002/acr.21896 23203795 PMC3935235

[2] Wolfe F, Ross K, Anderson J, Russell IJ, Hebert L. The prevalence and characteristics of fibromyalgia in the general population. Arthritis Rheum 1995; 38: 19–28. 10.1002/art.1780380104 7818567

[3] Borchers AT, Gershwin ME. Fibromyalgia: a critical and comprehensive review. Clin Rev Allergy Immunol 2015; 49: 100–151. 10.1007/s12016-015-8509-4 26445775

[4] Macfarlane GJ, Kronisch C, Dean LE, Atzeni F, Hauser W, Fluss E, et al. EULAR revised recommendations for the management of fibromyalgia. Ann Rheum Dis 2017; 76: 318–328. 10.1136/annrheumdis-2016-209724 27377815

[5] Clauw DJ. Fibromyalgia: a clinical review. JAMA 2014; 311: 1547–1555. 10.1001/jama.2014.3266 24737367

[6] Stejskal V, Ockert K, Bjorklund G. Metal-induced inflammation triggers fibromyalgia in metal-allergic patients. Neuro Endocrinol Lett 2013; 34: 559–565. 24378456

[7] Bjorklund G, Dadar M, Aaseth J. Delayed-type hypersensitivity to metals in connective tissue diseases and fibromyalgia. Environ Res 2018; 161: 573–579. 10.1016/j.envres.2017.12.004 29245125

[8] Marcusson JA, Lindh G, Evengard B. Chronic fatigue syndrome and nickel allergy. Contact Dermatitis 1999; 40: 269–272. 10.1111/j.1600-0536.1999.tb06061.x 10344482

[9] Marcusson JA. Contact allergies to nickel sulfate, gold sodium thiosulfate and palladium chloride in patients claiming side-effects from dental alloy components. Contact Dermatitis 1996; 34: 320–323. 10.1111/j.1600-0536.1996.tb02215.x 8807223

[10] Sabroe RA, Sharp LA, Peachey RD. Contact allergy to gold sodium thiosulfate. Contact Dermatitis 1996; 34: 345–348. 10.1111/j.1600-0536.1996.tb02220.x 8807228

[11] Bjorkner B, Bruze M, Moller H. High frequency of contact allergy to gold sodium thiosulfate: an indication of gold allergy? Contact Dermatitis 1994; 30: 144–151. 10.1111/j.1600-0536.1994.tb00695.x 8187513

[12] Tsuruta K, Matsunaga K, Suzuki K, Suzuki R, Akita H, Washimi Y, et al. Female predominance of gold allergy. Contact Dermatitis 2001; 44: 55–56. 10.1034/j.1600-0536.2001.440107-22.x 11156030

[13] Möller H, Björkner B, Bruze M. Clinical reactions to systemic provocation with gold sodium thiomalate in patients with contact allergy to gold. Br J Dermatol 1996; 135: 423–427. 10.1111/j.1365-2133.1996.tb01507.x 8949437

[14] Moller H, Larsson A, Bjorkner B, Bruze M, Hagstam A. Flare-up at contact allergy sites in a gold-treated rheumatic patient. Acta Derm Venereol 1996; 76: 55–58. 10.2340/00015555765558 8721495

[15] Svensson A, Moller H, Bjorkner B, Bruze M, Leden I, Theander J, et al. Rheumatoid arthritis, gold therapy, contact allergy and blood cytokines. BMC Dermatol 2002; 2: 2. 10.1186/1471-5945-2-2 11860615 PMC65540

[16] Pongpairoj K, Ale I, Andersen KE, Bruze M, Diepgen TL, Elsner PU, et al. Proposed ICDRG classification of the clinical presentation of contact allergy. Dermatitis 2016; 27: 248–258. 10.1097/DER.0000000000000222 27608064

[17] Ahnlide I, Ahlgren C, Bjorkner B, Bruze M, Lundh T, Moller H, et al. Gold concentration in blood in relation to the number of gold restorations and contact allergy to gold. Acta Odontol Scand 2002; 60: 301–305. 10.1080/00016350260248283 12418721

[18] Ahlgren C, Ahnlide I, Bjorkner B, Bruze M, Liedholm R, Moller H, et al. Contact allergy to gold is correlated to dental gold. Acta Derm Venereol 2002; 82: 41–44. 10.1080/000155502753600876 12013197

[19] Tsushima F, Sakurai J, Shimizu R, Kayamori K, Harada H. Oral lichenoid contact lesions related to dental metal allergy may resolve after allergen removal. J Dent Sci 2022; 17: 1300–1306. 10.1016/j.jds.2021.11.008 35784139 PMC9236887

[20] Torgerson RR, Davis MD, Bruce AJ, Farmer SA, Rogers RS 3rd. Contact allergy in oral disease. J Am Acad Dermatol 2007; 57: 315–321. 10.1016/j.jaad.2007.04.017 17532095

[21] Ahlgren C, Bruze M, Moller H, Gruvberger B, Axell T, Liedholm R, et al. Contact allergy to gold in patients with oral lichen lesions. Acta Derm Venereol 2012; 92: 138–143. 10.2340/00015555-1247 22170162

[22] Hopkins K, Antelmi A, Dahlin J, Olsson K, Svedman C, Astrand J, et al. Increased rates of gold and acrylate allergy in individuals with fibromyalgia tested with an extended dental patch test series. Acta Derm Venereol 2023; 103: adv22336. 10.2340/actadv.v103.22336 38078690 PMC10726376

[23] Bruze M, Hopkins K, Dahlin J, Olsson K, Astrand J, Svedman C, et al. Increased rates of fragrance allergy in fibromyalgia individuals tested with the Swedish baseline patch test series. J Eur Acad Dermatol Venereol 2023; 37: 104–113. 10.1111/jdv.18562 36018078

[24] Bruze M, Edman B, Bjorkner B, Moller H. Clinical relevance of contact allergy to gold sodium thiosulfate. J Am Acad Dermatol 1994; 31: 579–583. 10.1016/S0190-9622(94)70219-5 8089283

[25] Heller GZ, Manuguerra M, Chow R. How to analyze the visual analogue scale: myths, truths and clinical relevance. Scand J Pain 2016; 13: 67–75. 10.1016/j.sjpain.2016.06.012 28850536

[26] Bruze M, Andersen KE. Gold – a controversial sensitizer. European Environmental and Contact Dermatitis Research Group. Contact Dermatitis 1999; 40: 295–299. 10.1111/j.1600-0536.1999.tb06079.x 10385331

[27] Dr.Andersen KE, Jensen CD. Long-lasting patch reactions to gold sodium thiosulfate occurs frequently in healthy volunteers. Contact Dermatitis 2007; 56: 214–217. 10.1111/j.1600-0536.2007.01049.x 17343622

[28] Paulsen E, Andersen F, Vestergaard L, Andersen KE. Screening for gold sensitization in consecutive eczema patients: prevalence, relevance, and sources of exposure. Dermatitis 2019; 30: 222–226. 10.1097/DER.0000000000000477 31045934

[29] Fleming C, Lucke T, Forsyth A, Rees S, Lever R, Wray D, et al. A controlled study of gold contact hypersensitivity. Contact Dermatitis 1998; 38: 137–139. 10.1111/j.1600-0536.1998.tb05679.x 9536404

[30] Bruze M, Hedman H, Bjorkner B, Moller H. The development and course of test reactions to gold sodium thiosulfate. Contact Dermatitis 1995; 33: 386–391. 10.1111/j.1600-0536.1995.tb02072.x 8706395

[31] Moller H, Svensson A. Metal sensitivity: positive history but negative test indicates atopy. Contact Dermatitis 1986; 14: 57–60. 10.1111/j.1600-0536.1986.tb01154.x 3948511

[32] Liden C, Nordenadler M, Skare L. Metal release from gold-containing jewelry materials: no gold release detected. Contact Dermatitis 1998; 39: 281–285. 10.1111/j.1600-0536.1998.tb05942.x 9874018

[33] Svedman C, Gruvberger B, Dahlin J, Persson L, Moller H, Bruze M. Evaluation of a method for detecting metal release from gold; cysteine enhances release. Acta Derm Venereol 2013; 93: 577–578. 10.2340/00015555-1541 23529113

[34] Schaffran RM, Storrs FJ, Schalock P. Prevalence of gold sensitivity in asymptomatic individuals with gold dental restorations. Am J Contact Dermat 1999; 10: 201–206. 10.1097/01206501-199912000-00004 10594295

[35] Laeijendecker R, van Joost T. Oral manifestations of gold allergy. J Am Acad Dermatol 1994; 30: 205–209. 10.1016/S0190-9622(94)70018-4 8288779

[36] Vamnes JS, Morken T, Helland S, Gjerdet NR. Dental gold alloys and contact hypersensitivity. Contact Dermatitis 2000; 42: 128–133. 10.1034/j.1600-0536.2000.042003128.x 10727162

[37] Ekqvist S, Svedman C, Moller H, Kehler M, Pripp CM, Bjork J, et al. High frequency of contact allergy to gold in patients with endovascular coronary stents. Br J Dermatol 2007; 157: 730–738. 10.1111/j.1365-2133.2007.08119.x 17711524

[38] Tsiakiris G, Neely G, Lind N, Nordin S. Comorbidity in allergic asthma and allergic rhinitis: functional somatic syndromes. Psychol Health Med 2017; 22: 1163–1168. 10.1080/13548506.2016.1276606 28034321

[39] Balasubramaniam R, Laudenbach JM, Stoopler ET. Fibromyalgia: an update for oral health care providers. Oral Surg Oral Med Oral Pathol Oral Radiol Endod 2007; 104: 589–602. 10.1016/j.tripleo.2007.05.010 17964475

[40] Rhodus NL, Fricton J, Carlson P, Messner R. Oral symptoms associated with fibromyalgia syndrome. J Rheumatol 2003; 30: 1841–1845. 12913944

