# Supplementary material for: Clinical Relevance of Contact Allergy to Gold Sodium Thiosulphate in Fibromyalgia
Source: Acta Derm Venereol. 2025 Mar 27;105:42463. doi: 10.2340/actadv.v105.42463 (PMC11971835; doi:10.2340/actadv.v105.42463)
Supplement: Clinical Relevance of Contact Allergy to Gold Sodium Thiosulphate in Fibromyalgia [file ActaDV-105-42463-s1.pdf]

Supplementary material has been published as submitted. It has not been copyedited, or typeset by Acta Dermato-Venereologica

Appendix S1: Participant questionnaire: fibromyalgia and contact allergy

1. Approximately how long have you had fibromyalgia?
   (1= up to 6 months, 2= up to 1 year, 3= up to 3 years, 4= up to 10 years, 5= over 10 years)
2. Who did you receive your diagnosis of fibromyalgia from?
   (1= Rheumatologist/pain specialist/doctor at hospital clinic, 2= general practitioner, 3= other type of doctor, 4= other healthcare professional)
3. Do you have high blood pressure?
   (1=yes, 2= don't know, 3= no)
4. Do you have heart disease?
   (1=yes, 2= don't know, 3= no)
5. If yes, what type of heart disease
   (Answer with free text)
6. Do you have rheumatoid arthritis?
   (1=yes, 2= don't know, 3= no)
7. Do you have any other rheumatological inflammatory illness?
   (1=yes, 2= don't know, 3= no)
8. If yes, what type of rheumatological inflammatory illness?
   (Answer with free text)
9. Do you have systemic lupus erythematosus (SLE)?
   (1=yes, 2= don't know, 3= no)
10. Do you have depression/anxiety/ other psychiatric illness?
    (1=yes, 2= don't know, 3= no)
11. Do you have any other illnesses?
    (Answer with free text)
12. Are you prescribed any steroid treatment (tablet form eg) prednisolone, betapred)
    (1=yes, 2=no)
13. If yes, which steroid preparation and dose?
    (Answer with free text)
14. Are you undergoing treatment with immunnosuppressants?
    (1=yes, 2=no)
15. Do you use or have you used anti-inflammatory gel eg) ibuprofen, diclofenac gel?
    (1=yes, 2= don't know, 3= no)
16. If yes, which preparation?
    (Answer with free text)
17. Do you have asthma or hay fever (rhinitis)?
    (1=yes, 2= don't know, 3= no)
18. Do you have or have previously had psoriasis?
    (1=yes, 2= don't know, 3= no)
19. Do you have or have previously had eczema?
    (1=yes, 2= don't know, 3= no)
20. Do you have any other type of skin disease or condition?
    (Answer with free text)

21. Do you use sunscreen?
    (1=yes, 2=no)
22. Have you experienced any problems/side effects from sunscreen?
    (1=yes, 2=no)
23. Are you sensitive to perfumes/scents?
    (1=yes, 2=no)
24. Have you experienced any problems/side effects from exposure to perfumes?
    (1=yes, 2=no)
25. Have you experienced any airway/breathing problems from exposure to perfumes?
    (1=yes, 2=no)
26. Are you sensitive to vehicle exhaust fumes?
    (1=yes, 2=no)
27. Are you sensitive to flower scents?
    (1=yes, 2=no)
28. Have you ever had patch testing for contact allergy?
    (1=yes, 2= don't know, 3= no)
29. Did the patch test show any allergies?
    (1=yes, 2= don't know, 3= no)
30. Which allergies?
    (Answer with free text)
31. How much pain have you had in the last week due to your fibromyalgia?
    (VAS=0mm no pain-100mm maximum amount of pain)
32. How have you felt generally over the last week, with respect to your fibromyalgia?
    (VAS= 0mm completely well-100mm as bad as I can imagine)
33. How tired have you been over the last week due to your fibromyalgia?
    (VAS= 0mm no tiredness-100mm worst possible tiredness)
34. Have you ever experienced any problems with itching, redness or swelling upon direct skin contact with gold objects such as earrings or rings?
    (1=yes, 2= don't know, 3= no)
35. Have you ever experienced any problems with itching, redness or swelling upon direct skin contact with other metal jewellery or metal objects?
    (1=yes, 2= don't know, 3= no)
36. Do you have pierced ears?
    (1= yes, 2=no)
37. Do you have any other piercings?
    (1=yes, 2=no)
38. Do you have, or have you had any gold dental restorations, bridge, etc?
    (1=yes, 2= don't know, 3= no)
39. Do you have any oral problems or symptoms? (for example, stinging, swelling, redness, sores/ulcers?)
    (1=yes, 2= don't know, 3= no)
40. Do you have any genital symptoms? (For example swelling, redness, sores/ulcers?)
    (1=yes, 2= don't know, 3= no)
41. Do you smoke?
    (1=yes, 2=no)
42. Do you use snuff (snus)?

(1=yes, 2=no)

43. Do you use chewing gum regularly (all types)?
    (1=yes, 2=no)
44. Do you work, or have you worked with gold-containing material (eg) goldsmith)?
    (1=yes, 2= don't know, 3= no)
45. If yes, which occupation and when?
    (Answer with free text)
46. Do you work, or have you worked with nickel-containing material?
    (1=yes, 2= don't know, 3= no)
47. If yes, which occupation and when?
    (Answer with free text)
48. Do you use, or have you used medication which contains gold?
    (1=yes, 2= don't know, 3= no)
49. If yes, which medication and when?
    (Answer with free text)

APPENDIX S2.

List of allergens in the Swedish baseline series for patch testing and the extended dental series.

Swedish baseline series	Dental series
Potassium dichromate	Methyl methacrylate
p-Phenylene diamine	Triethylene glycol dimethacrylate
Thiuram mix	Urethane dimethacrylate
Neomycin sulfate	Ethylene glycol dimethacrylate
Cobalt chloride hexahydrate	BIS-GMA[a]
Quaternium 15	N,N-Dimethyl-4-toluidine
Nickel sulfate hexahydrate	2-Hydroxy-4-methoxy-4-methylbenzophenone
Quinoline mix	1,4-Butanediol dimethacrylate
Colophony	BIS-MA[b]
Paraben mix	Elemental mercury 0.5%
Black rubber mix	Sodium tetrachloro palladate
Sesquiterpene lactone mix	2-Hydroxyethyl methacrylate
Mercapto mix	N-Ethyl-p-toluenesulfonamide
Epoxy resin	4-Tolyldiethanolamine
Myroxolon pereirae	Copper sulfate
p-ter-Butylphenol formaldehyde resin	Methylhydroquinone
Fragrance mix II	Camphorquinone
Formaldehyde	Dimethylaminoethyl methacrylate
Fragrance mix I	1,6-Hexanediol diacrylate
Phenol formaldehyde resin	Drometrizole
Diazolidinyl urea	Tetrahydrofurfuryl methacrylate
Methylchloroisothiazolinone/	Elemental tin
methylisothiazolinone	Elemental titanium
Amerchol L 101	Calcium titanate
Caine mix II	Silver sulphate
Lichen acid mix	Ammonium hexachloroplatinate
Tixocortol-21-pivalate	Glutar aldehyde
Textile dye mix	Titanium nitride
Budesonide	Elemental mercury 1.6%
Methyldibromo glutaronitrile	Sodium metabisulfite

Methylisothiazolinone	BIS-EMA[c]<br>Thiomersal<br>Aluminium chloride hexahydrate<br>Gold sodium thiosulfate 2.0%<br>Canada balsam<br>Eugenol<br>Carvone<br>Cinnamal<br>Hydroxides of linalool<br>Hydroxides of limonene

[a]BIS-GMA: bisphenol A glycerolate dimethacrylate; [b]BIS-MA: bisphenol A dimethylacrylate. [c]BIS-EMA: 2,2-bis (4-(2-methacryloxyethoxy)phenyl)propane.
